# From Mild to Mayhem: A Life-Threatening Exacerbation of Mild Asthma

**DOI:** 10.7759/cureus.70558

**Published:** 2024-09-30

**Authors:** Kristina Brown, Lorraine Anderson

**Affiliations:** 1 Internal Medicine-Pediatrics, University of California Los Angeles, Los Angeles, USA; 2 Allergy and Immunology, University of California Los Angeles, Los Angeles, USA

**Keywords:** exacerbation, inhaled corticosteroid (ics), mild asthma, updated guidelines on asthma, when to refer

## Abstract

This case illustrates an example of a patient with mild asthma who experiences a severe life-threatening exacerbation. This patient’s experience is one of many that challenges the common misperception that mild, well-controlled asthma is not at risk for severe life-threatening exacerbations. The case exploration highlights the data fueling the new 2019/2020 Global Initiative for Asthma (GINA) guidelines, pharmaco-inequity, and barriers to healthcare access which can increase the risk for poor asthma outcomes. The graphic illustrates when patients with asthma, including those with mild asthma, may benefit from referral to an allergist-immunologist. Overall, this case underscores the importance of bridging the gap between generalists and allergist-immunologists to better serve an oft-overlooked patient population.

## Introduction

Asthma is characterized by airway hyperreactivity, inflammation, and mucus production which results in airway obstruction and limitation in airflow. Exacerbations can have multiple triggers including medications, viral infections, allergen/pollutant exposure, variations in the weather, stress, and physical exercise. Risk factors for worse asthma outcomes include incorrect inhaler technique, decreased medication compliance, high use of short-acting beta2-agonist (SABA), lack of prescribed inhaled corticosteroid (ICS), anxiety, allergens/pollutants, lower socioeconomic status, poor lung function (forced expiratory volume exhaled in the first second (FEV1) < 60%), history of intubation or having one or more severe exacerbations in the past year, among others [1].

Globally, asthma affects about 340 million people, of which approximately 50-75% have mild asthma [2]. The diagnosis of asthma is based on impairment, such as symptom frequency and spirometry, and risk, which includes exacerbation and intubation history. Mild asthma includes both mild intermittent asthma and mild persistent asthma. Mild intermittent asthma is delineated by symptoms occurring fewer than 2 days per week, 2 or fewer nighttime awakenings monthly, no activity limitations, normal FEV1 between exacerbations, FEV1 greater than 80% predicted, and normal FEV1/forced vital capacity (FVC) normal, and one or fewer exacerbations requiring steroids per year. Mild persistent asthma is characterized by symptoms occurring more than 2 days per week but not daily, 3-4 nighttime awakenings per month, minor limitation to normal activity, FEV1 greater than 80% predicted, FEV1/FVC normal, and exacerbations requiring steroids occurring more than twice per year [1]. About one in five patients with mild asthma have had an asthma-related hospitalization or severe exacerbation in the previous year. Though such patients experience lower rates of exacerbation individually, they comprise 30-50% of all asthma exacerbations requiring emergency care [2].

This case challenges the common misperception that mild, well-controlled asthma is not at risk for severe life-threatening exacerbations.

## Case presentation

A 19-year-old female with a history of seasonal allergies, anxiety, and mild asthma presented to the emergency department with dyspnea for five days. She reported using an albuterol inhaler infrequently but increased it to every three hours for the past two days with minimal relief. She delayed seeking care as she was studying for college exams. She denied fever, upper respiratory infection, and sick contacts and was up to date on immunizations including the COVID-19 vaccine. She had not taken any other medications and last visited her primary care doctor years ago. Her most common trigger for asthma exacerbation was viral infections and her last exacerbation was one year ago due to the COVID-19 virus. Four years ago, she was intubated for a severe asthma exacerbation triggered by a viral infection. The patient’s medical record did not indicate recent lung function tests or any prior allergy testing. She had no history of consultation with Allergy Immunology or Pulmonary. She reported that in her usual state of health, she was able to complete her activities without restriction, used albuterol up to twice a week, and felt that her asthma was well controlled. She does not smoke or vape.

In the emergency department, her vitals were normal. A comprehensive metabolic panel and complete blood count were reassuring and showed no signs of infection. She had no documented total IgE or peripheral eosinophil count. Chest X-ray was negative for pneumonia, but showed hyperinflation of the lungs. She was given three treatments of nebulized ipratropium bromide/albuterol, and IV methylprednisolone and observed for two hours. Her exam was notable for poor aeration, wheezing, and tachypnea despite the treatments, so she was given additional nebulized ipratropium bromide/albuterol, observed for one hour, and admitted for further management. Soon after, she developed increased work of breathing, difficulty speaking due to shortness of breath, and accessory muscle use. Her pulmonary exam continued to demonstrate poor aeration and diffuse wheezing. She was treated with IV methylprednisolone, IV magnesium, and continuous nebulized albuterol. Later that evening she developed respiratory distress with hypoxemia. Venous blood gas showed respiratory acidosis and she was treated with bilevel positive airway pressure. However, her increased work of breathing and respiratory acidosis persisted, requiring intubation. Her clinical picture and diagnostics were consistent with severe life-threatening asthma exacerbation. She was transferred to the intensive care unit for continued management, including ventilatory support and potassium chloride infusions. She was extubated the next day and discharged the following day on a course of prednisone and provided with an ICS-long-acting β2-agonist (LABA), with a scheduled follow-up with her primary care provider within two weeks.

## Discussion

The patient in our vignette had several risk factors for poor asthma outcomes: high SABA use, no prescribed ICS, history of anxiety, exposure to air pollution, and history of intubation. Additionally, our patient’s airway inflammation did not respond after initial treatment with systemic corticosteroids, and nebulized albuterol and ipratropium bromide. This coupled with the degree of airflow limitation, lack of consistent follow-up with primary care, and her living situation as a student with no history of evaluation by an Allergist or Pulmonologist helped to inform the clinical decision to admit the patient. Currently, there are no national guidelines to help guide the identification of patients who should be kept for overnight observation versus hospital admission versus discharge to home.

Decades ago, asthma was considered a disease mainly of bronchoconstriction. Previous guidelines for mild asthma recommended first-line, as-needed SABA alone, which induces bronchodilation. Newer studies show that SABA inhibits early, but not late asthmatic responses such as bronchoconstriction and airway hyper-responsiveness. It is now recognized that rising inflammation is the underlying cause of symptoms and exacerbations in all severities of asthma. Furthermore, data shows a paradigm shift toward recognizing that unpredictable rises in airway inflammation significantly contribute to asthma exacerbations, not just bronchoconstriction [2]. SABA therapy alone treats bronchoconstriction but fails to address airway inflammation and can worsen inflammation; it is associated with increased airway inflammatory cells and increased fractional exhaled nitric oxide (FeNO) levels [3]. Use of SABA alone is associated with a higher risk of exacerbations and diminished lung function. Additionally, SABA overuse is associated with an increased risk of severe exacerbations and asthma-related death. Studies show that airway inflammation impacts most asthma patients, regardless of symptom frequency.

Fundamentally, airway inflammation must be addressed in all patients with asthma to decrease impairment and reduce the risk of exacerbation. In 2019/2020, the Global Initiative for Asthma (GINA) produced updated, evidence-based recommendations that ICS-containing treatment is integral to first-line treatment for all patients with asthma. Low-dose ICS reduces asthma hospitalizations, death, and severe exacerbations, and improves lung function if started early [1]. Patients with mild asthma who are not on a maintenance inhaler are recommended to use an ICS-containing rescue treatment (ICS/LABA or ICS/SABA) rather than SABAs as this option reduces the risk of severe exacerbations, prolongs the time to first severe exacerbation, and reduces impairment by increasing the probability of well-controlled asthma.

Although the patient described was seen years after the change in GINA guidelines, she had not seen a primary care physician for several years. Her suboptimal asthma treatment and delayed presentation to medical care were potentially impacted by her busy college schedule and challenges transitioning from pediatric to adult primary care. Studies show that young adults with asthma face particular challenges in access to asthma care including fewer follow-ups and greater lapses in prescription medications. Additionally, mid to late adolescents (ages 16-20) have expressed asthma burden and feelings of inadequate symptom self-management. Some studies suggest that healthcare providers and parents may not be sufficiently preparing this age group to successfully transition care which can further the gaps in asthma control [4].

Given this and the GINA guideline updates, it is important to reconnect patients with dedicated visits for asthma education and management and prescribe ICS-containing reliever therapy (ICS/LABA or ICS/SABA). Options include budesonide/formoterol, mometasone/formoterol, and budesonide/albuterol. Despite the options available, disparities in pharmaco-equity impact whether patients can actually afford needed therapies. Budesonide-formoterol, for instance, can cost 20 times more than albuterol, depending on the insurance company. Rather than be prescribed combined therapy, some patients have two separate inhalers which can be challenging to manage. Furthermore, many US insurance companies will limit or deny ICS-LABA for reliever use. While the patient of interest was provided an ICS-LABA on discharge, it is unclear if she can sustainably afford further refills [5].

Overall, asthma exacerbations are often the result of uncontrolled inflammation which can be unpredictable. While it may not be intuitive to send patients with mild asthma to an allergist-immunologist, this subgroup may otherwise be neglected. The figure below illustrates when patients with asthma, including those with mild asthma, may benefit from referral to an allergist-immunologist [6]. Studies show that patients with asthma have lower odds of asthma hospitalization and SABA overuse, greater knowledge of self-managing symptoms, and higher asthma-related quality of life when seeing specialists versus generalists [7]. In a study of children with two or more ED visits or one or more hospitalizations for asthma within the previous year, those with generalist follow-up were twice as likely to require hospitalization and 40% more likely to visit the ED for asthma as compared to patients managed in an allergy clinic [8]. Some guidelines suggest that patients with asthma should be seen every 1-6 months to monitor control. Patients in the midst of gaining more control of their symptoms should be seen every 2-6 weeks until their asthma is well controlled [9].

**Figure 1:**
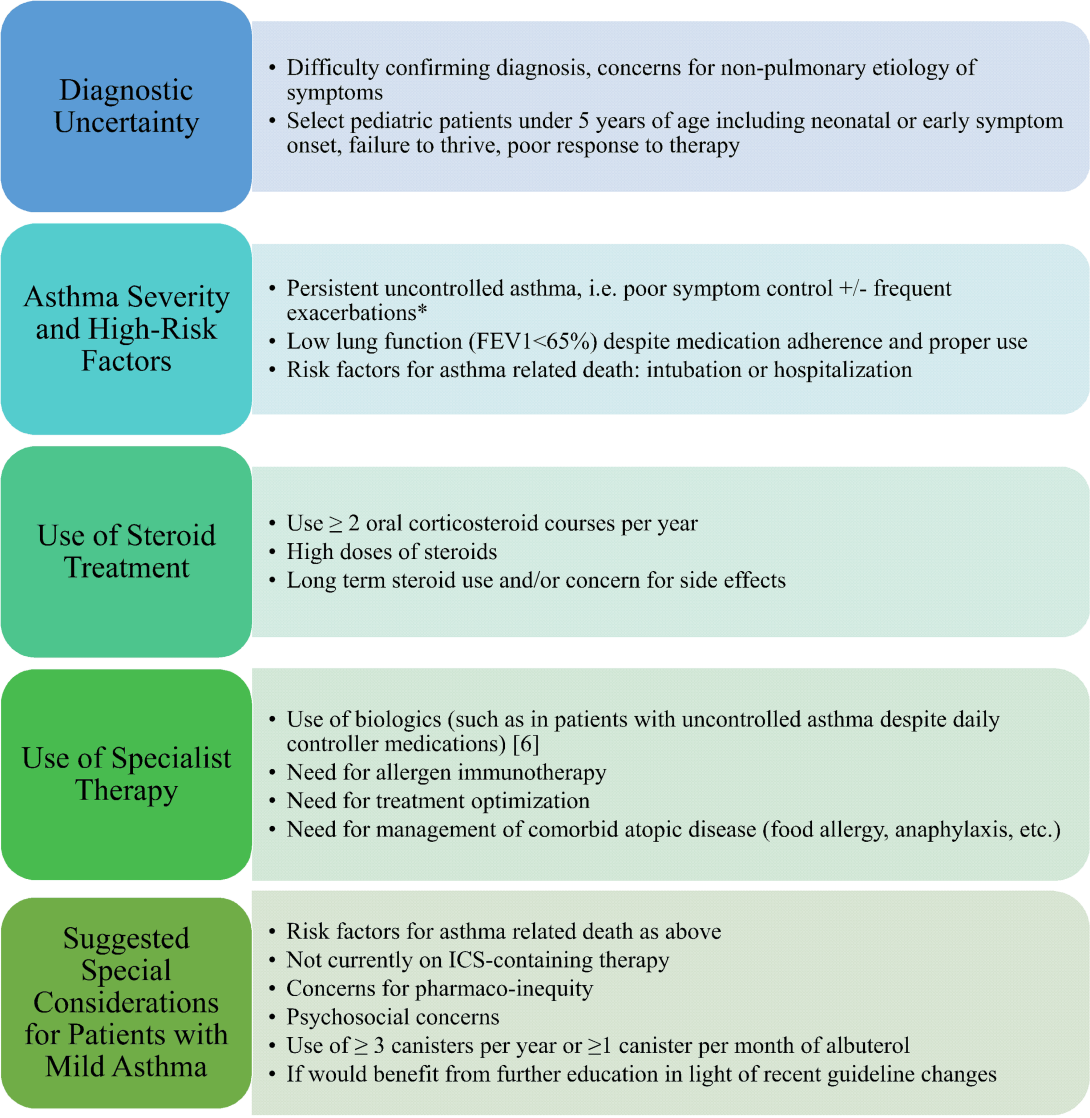
Bridging the Gap: Considerations for Referral of Patients with Asthma to Specialist Care *Poor symptom control includes frequent symptoms, frequent reliever use, activity limitations, and night awakenings due to asthma. Frequent exacerbations are defined as 2 or more per year requiring oral steroids, or at least 1 exacerbation requiring hospitalization FEV1: forced expiratory volume exhaled in the first second; ICS: inhaled corticosteroid

## Conclusions

Referral of patients with asthma to an allergist should not be restricted to those with severe asthma. Patients benefit from regular, dedicated time to address inhaler technique, adjust medications, and re-evaluate pulmonary function tests, risk factors and impairment, and asthma action plans. It is well known that busy primary care offices face heavy demands to address multiple medical issues under significant time constraints. Additionally, young adults with asthma can be at risk of falling through the cracks, and disparities in the costs of ICS-containing therapies are yet another challenge to overcome. In conjunction with generalists, allergists can offer additional expertise, time, and education and thus be a valuable resource in co-managing patients with asthma across the disease spectrum.
